# Supplementary material for: Bioremediation Potential of Native Bacillus sp. Strains as a Sustainable Strategy for Cadmium Accumulation of Theobroma cacao in Amazonas Region
Source: Microorganisms. 2022 Oct 25;10(11):2108. doi: 10.3390/microorganisms10112108 (PMC9698815; doi:10.3390/microorganisms10112108)
Supplement: Supplementary file 1 [file microorganisms-10-02108-s001.zip › microorganisms-1922463-supplementary tables.pdf]

Supplementary Table S1. Cadmium survivor characterization by MIC of bacteria isolates associated with cacao.

Sampling site	Altitude (msnm)	Coordinates	pH	Soil Cd^{2+} (ppm)	Isolates	Cadmium concentration (ppm)								
						**0**	**50**	**100**	**150**	**200**	**250**	**300**	**350**	**400**
Aramango-O	515	5º25`49"S-78º26`43"W	5.9	1.97	9	9	9	9	9	9	9	9	1	-
Aramango-F	859	5º26`0.5"S-78º25`20"W	6.6	3.54	14	14	14	14	14	14	13	13	1	-
La Peca-H	764	5º37`26"S-78º26`27"W	6.9	1.52	10	10	10	10	10	10	10	10	7	-
La Peca-J	981	5º35`14"S-78º26`40"W	5.0	1.47	8	8	8	8	8	8	8	7	7	-
La Peca-T	1033	5º37`37"S-78º25`22"W	6.7	1.62	10	10	10	9	9	9	9	9	7	-
Copallin-V	785	5º41`32"S-78º24`19"W	7.9	2.61	9	9	9	9	9	8	8	7	1	1
Copallin-S	801	5º41`30"S-78º24`15"W	7.9	2.18	12	12	12	12	12	12	12	12	2	1
Copallin-A	820	5º40`53"S-78º24`40"W	7.9	1.46	12	12	12	12	12	12	5	5	0	-
Copallin-Z	830	5º41`17"S-78º24`13"W	8.1	2.1	14	14	14	14	14	14	14	13	1	-
Copallin-C	894	5º40`53"S-78º24`17"W	7.0	1.59	9	9	9	9	9	8	8	8	5	-
Copallin-M	917	5º40`44"S-78º24`18"W	6.9	2.6	10	10	10	10	9	9	9	8	7	-
Copallin-E	931	5º40`29"S-78º24`32"W	6.1	2.64	9	9	9	9	9	9	9	9	3	-
Copallin-R	1176	5º39`01"S-78º23`17"W	7.6	2.14	12	12	12	12	12	12	12	12	9	1
		**TOTAL**			**138**	**138**	**138**	**137**	**136**	**134**	**126**	**122**	**51**	**3**

Isolates: The values indicate the total number of isolation per sample site. **Cadmium concentration**: The values indicate the number of tolerant isolation obtained at this concentration.

Supplementary Table S2. PGPR attributes characterization

Strain	PGPR atributes		
	**SE (Zn)%**	**SI (P)**	**Sid (cm^2)**
**V3C3**	313.00 ± 27.50	1.11 ± 0.05[a]	3.71 ± 1.92
**R1C2**	365.99 ± 27.50	1.57 ± 0.05[b]	Nd
**S1C2**	333.54 ± 27.50	1.09 ± 0.05[a]	2.62 ± 1.27

The values represent the means and standard deviation. Nd: No determined

SE: Zn Solubilization efficiency, SI: Phosphorus solubilization Index,

Sid: Siderophore production area
