# Supplementary figures and images for: Bioremediation Potential of Native Bacillus sp. Strains as a Sustainable Strategy for Cadmium Accumulation of Theobroma cacao in Amazonas Region
Source: Microorganisms. 2022 Oct 25;10(11):2108. doi: 10.3390/microorganisms10112108 (PMC9698815; doi:10.3390/microorganisms10112108)

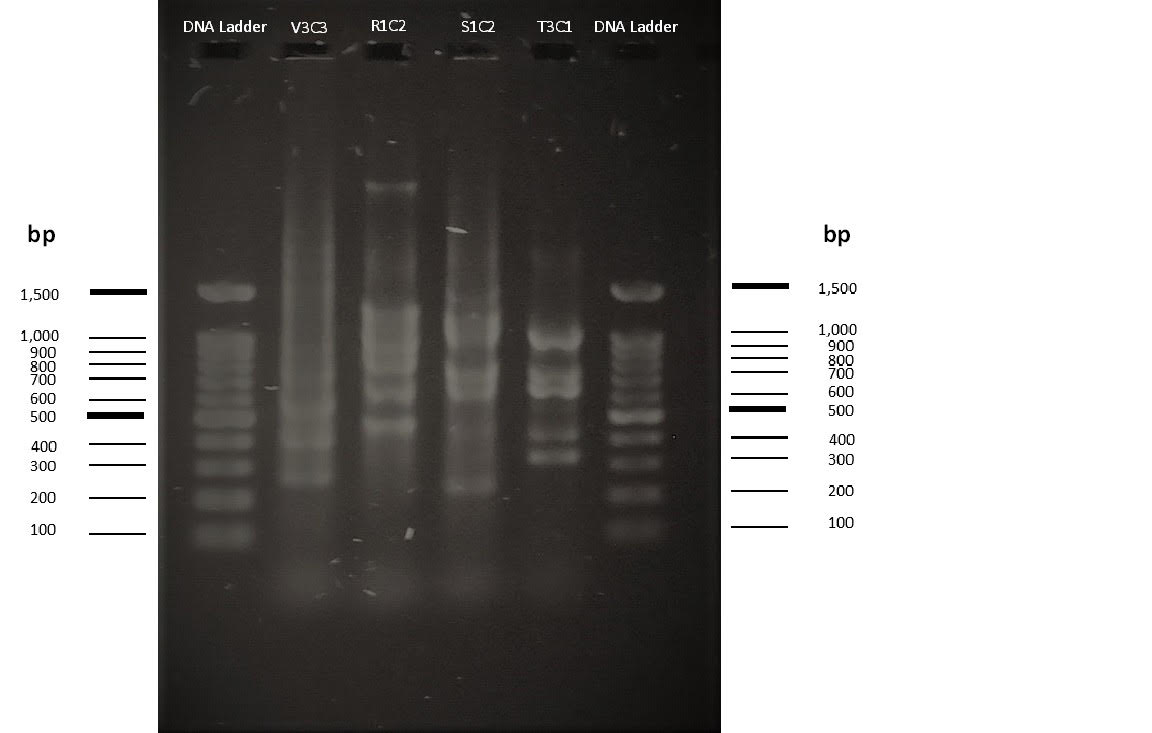

Supplement: Supplementary file 1 [file microorganisms-10-02108-s001.zip › supplementary figure S1.jpg]

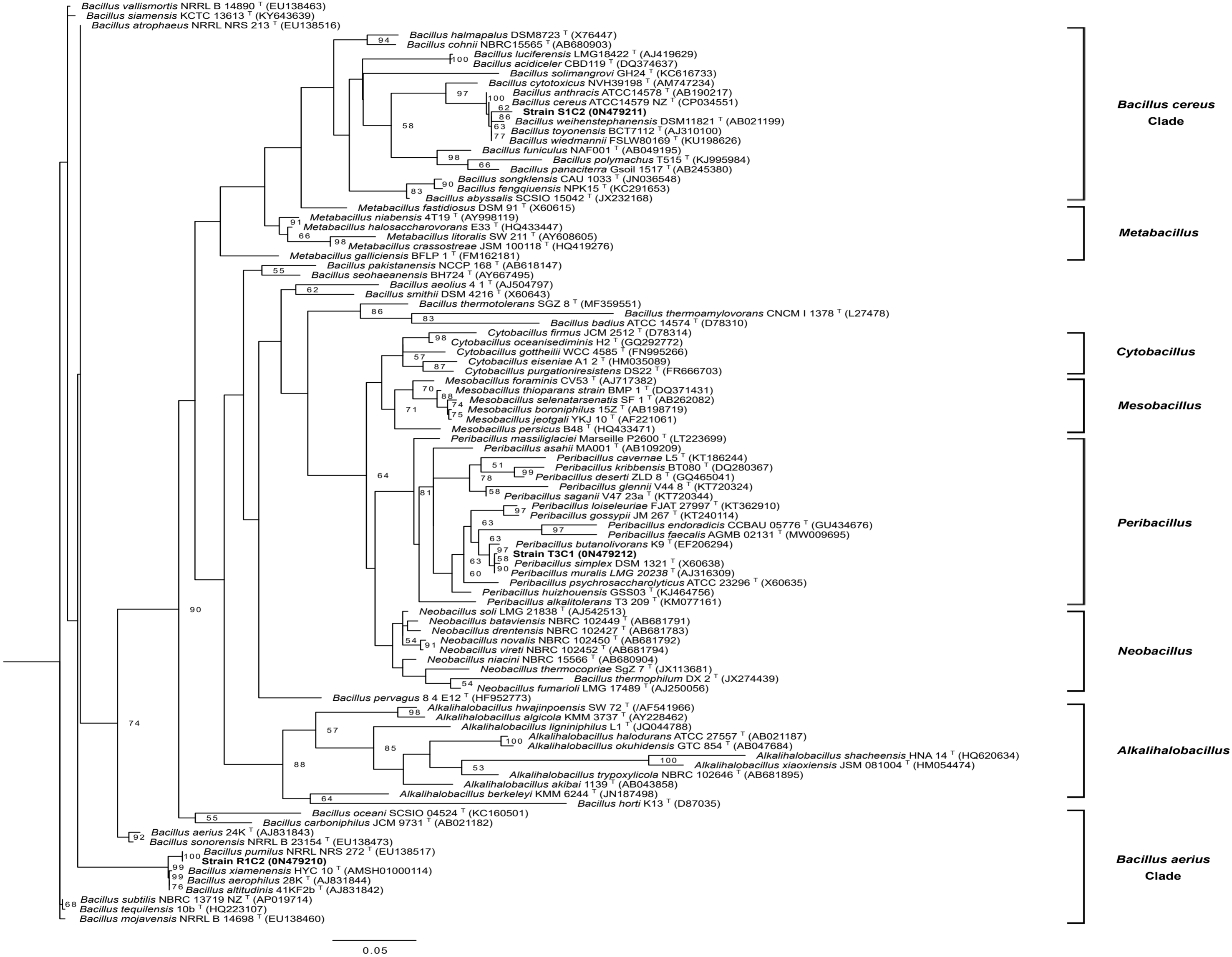

Supplement: Supplementary file 1 [file microorganisms-10-02108-s001.zip › Supplmentary figure S2.pdf]
